# The study about the difference of extra cellular volume calculated with T1mapping in dilated cardiomyopathy with and without late gadolinium enhancement

**DOI:** 10.1186/1532-429X-17-S1-P306

**Published:** 2015-02-03

**Authors:** Kunihiko Teraoka, Yoshinori Suzuki, Yoshiaki Komori

**Affiliations:** 1 Dept of Cardiology, Tokyo Medical Univerity Hachioji Medical Center, Tokyo, Japan; 2 Department of Radiology, Tokyo Medical University Hachioji Medical Center, Tokyo, Japan; 3 Kyoto university, Kyoto, Japan

## Background

It is well known that the DCM patients having Late Gadolinium Enhancement at Mid-layer of LV septal wall (Mid-LGE) shows poor prognosis compared to the case not having Mid-LGE

However, about the other histopathological differences by the existence of Mid-LGE, it is not clear.

On the other hand, in recent years, it became possible to calculate ECV from measurement of T1 value of the myocardium before and after of enhanced imaging which used the Modified Look-Locker Inversion Recovery(MOLLI) method, and the usefulness in the quantitative evaluation of histopathological changes, such as diffuse fibrosis observed in hypertrophic cardiomyopathy, has been reported.

We will study about the difference of extra cellular volume(ECV) calculated with T1mapping in DCM with and without LGE

## Methods

Twenty-one cases diagnosed as DCM by cardiac MRI and eleven normal controls from October, 2012 to December, 2013 were examined.

T1 value of each area of midventricular slice and blood pool in the left ventricle was measured for every case, and T1 value of a septum area, areas other than a septum, and all the sections were computed.

In measurement of T1 value, MOLLI 8-2 supported by Siemens, Erlangen, Germany was used.

ECV was computed from T1 value and hematocrit value of each area on before and after enhancement, and were taken as the median value of each measured value of a septum area, areas other than a septum, and all the sections were calculated.

## Results

1) DCM was divided into LGE (+) group ;10 cases and DCM LGE(-) ;11 cases in the visual assessment of the LGE CMR imaging.

2) Between two DCM groups with and without LGE, age, a hematocrit, EF and other cardiac parameters, significant differences were not recognized.

3) Native T1 value of the LGE(+) group was intentionally high only in the septal area as compared with LGE(-) group and normal group(975.3+/-35.3ms,958.5+/-38.6ms,910+/-14.8ms, retrospectively, P=0.024).

Native T1 Value of areas other than a septum and a total section, there was no significant difference between 3 groups.

4) The median value of ECV in total slice of LGE (+) group was significantly higher than that of LGE(-) group and the normal group(34.4+/-2.2%,26.6+/-2.7%,27.7+/-1.9%, retrospectively, P=0.01). The median value of ECV of LGE (+) was higher than that of LGE(-) group and normal group even in the areas other than a septum(32.5+/-2.4%,27.4+/-2.4%,26.4+/-1.7%, retrospectively, P=0.005).

## Conclusions

The median value of ECV of LGE(+) group was higher than that of LGE(-) group even in the areas other than a septum not showing LGE. Calculation of ECV by T1mapping may be used for evaluation of the stage of illness with DCM.

## Funding

None.

